# Fibula allograft propping as an effective treatment for early-stage osteonecrosis of the femoral head: a systematic review

**DOI:** 10.1186/s13018-020-01730-6

**Published:** 2020-06-03

**Authors:** Ju’an Yue, He Gao, Xiaozhong Guo, Randong Wang, Bing Li, Qiang Sun, Wangyan Liu, Jiao Chen, Yingnan Li

**Affiliations:** 1 Department of Joint Surgery, Aviation General Hospital, Courtyard 3, AnwaiBeiyuan, Chaoyang District, Beijing, China; 2 Disinfection Supply Division, Aviation General Hospital, Beijing, China

**Keywords:** Fibula allograft, Systematic review, Femoral head, Osteonecrosis

## Abstract

**Background:**

Osteonecrosis of the femoral head (ONFH) causes severe hip dysfunction. Left untreated, 80% of patients experience femoral head collapse, and 65–70% of patients require total hip arthroplasty (THA). Therefore, effective treatment is very important for ONFH.

**Objective:**

To examine the effectiveness of fibula allografting for the treatment of early-stage ONFH

**Methods:**

A systematic review was conducted by searching PubMed, EMBASE, and Web of Science databases using “avascular necrosis” or “ischemic necrosis” or “osteonecrosis” and “femoral head” and “fibula*,” and checking the references of primary articles and reviews. Two independent authors completed the study selection separately. We extracted the following details from each article: characteristics of the patients, clinical efficacy evaluation (Harris hip score [HSS], radiographic outcomes, the rate of conversation to total hip arthroplasty [THA], and adverse effects).

**Results:**

A total of 213 articles were selected from PubMed (*n* = 45), EMBASE (*n* = 77), Web of Science (*n* = 203), and other sources (*n* = 10). After checking the articles, five articles were included in the final analysis. The average age of patients involved in this review was 34.48 years. The studies investigated fibula allografts to treat ONFH in 394 hips with a mean follow-up of 49.06 months. HHS was improved from 62.73 to 86.94. Radiographic progression was found in 33.66% of hips. The failure rate of head-saving surgery by THA was 14.5%. No patients had serious postoperative complications.

**Limitations:**

The number of articles included in the study was small, and all studies were single-center studies. Most studies were retrospective with a low level of evidence. Surgical procedures were not identical with different follow-up times.

**Conclusion:**

Although there are some limitations to our approach, this systematic review supports fibula allografting as a simple, effective treatment for early-stage ONFH, which presents less postoperative complications, and has a satisfactory clinical effect. We consider it to be worthy of promotion as a therapy for ONFH.

## Introduction

Osteonecrosis of the femoral head (ONFH), which is usually divided into traumatic and non-traumatic ONFH, is also known as aseptic necrosis or avascular necrosis of the femoral head [1, 2]. Its cause is the death of osteocytes under the action of complex factors, which induces bone changes, subchondral bone fracture collapse, and changes in the shape and function of the femoral head. Traumatic ONFH is common in femoral neck fractures and hip dislocation. Hormone use has become the leading cause of non-traumatic ONFH [3]. Hormones are widely used for the treatment of rheumatoid arthritis, diffuse connective tissue disease, and immune-related allergic diseases, among which 5–40% of patients develop ONFH after using large doses of hormones. ONFH mainly occurs in young patients with a high disability rate [4]. If the patient’s condition is not detected in a timely manner and treated effectively, 80% of patients will experience femoral head collapse [5, 6], and about 65–70% of patients require total hip arthroplasty (THA) to improve limb functions [7].

THA as a mature and classic orthopedic treatment has made great achievements in the treatment of hip joint disease [8]. However, young and middle-aged patients with ONFH using joint replacements have a greater incidence of complications such as infection, loosening, and long-term revision surgery [9]. Therefore, the main purpose of the treatment of early-stage ONFH in young adults should be to improve symptoms and functions, preserve the femoral head as much as possible, delay the time of joint replacement, and finally avoid artificial joint replacement [10].

There are many femoral head-preserving procedures, including restricted weight bearing protocols [11], core decompression [12], allogeneic bone compression and bone grafting [13], vascular bone flap implantation [14, 15], non/free-vascularized bone grafting [16], and osteotomy [10]. Non-vascularized bone grafting is simple, easy to perform, and more appealing [17]. Vascularized bone grafting is a demanding technique with high technical requirements and a long operative time [18], which is difficult to promote clinically. Fibula allografting provides strong support and is a simple operation without donor site morbidity, which has achieved good clinical effects in the treatment of early-stage ONFH.

Fibula allografting is becoming an increasingly popular technique for ONFH treatment. The majority studies of fibula allografting to the femoral head have been at single institutions and are hardly universal. We performed this systematic review to examine the effectiveness of fibula allografting for the treatment of early-stage ONFH, particularly improving hip functions through Harris hip scoring (HHS), block imaging progression, and preventing the conversion to THA.

## Materials and methods

A systematic review was conducted by searching PubMed, EMBASE, and Web of Science databases in accordance with the PRISMA (Preferred Reporting Items for Systematic reviews and Meta analyses) guidelines. This review did not require ethical approval because it did not involve any processing of individual patient data. Search terms included “avascular necrosis” or “ischemic necrosis” or “osteonecrosis” and “femoral head” and “fibula*.” In addition, we checked the references of primary articles and reviews to avoid missing relevant articles. The articles included in our review were limited to English only and published at any time. Unpublished articles were not included in this review. Two independent authors separately completed the study selection. Any difference in opinions was resolved by discussion.

Criteria for inclusion in the review were as follows: the patients included in the study were patients with ONFH; randomized or nonrandomized clinical trials were included; ONFH patients were treated by a fibula allograft or compared a fibula allograft with other treatments; the article contained the required data. The exclusion criteria were animal studies, case reports, systematic reviews, and reports in languages other than English.

### Data acquisition

We extracted the following details from each article: the first author’s name, publication year, demographic characteristics (number of hips, ONFH stage, sex, age, and follow-up time), clinical efficacy evaluation HHS, radiographic outcomes, the rate of conversation to THA, and adverse effects. The weighted average was calculated based on the total number of patients in each study to control for the size of the different cohorts.

## Results

**Fig. 1:**
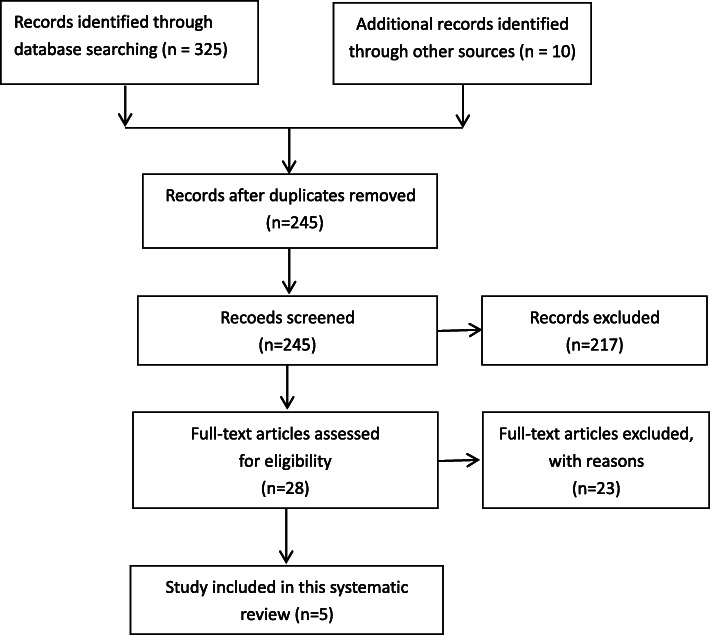
Flowchart of the selection process

A total of 335 articles were selected from PubMed (*n* = 45), EMBASE (*n* = 77), Web of Science (*n* = 203), and other sources (*n* = 10). As illustrated in Fig. 1, after duplicate checking, title and abstract screening, and full text screening, five articles were ultimately included in the final analysis (Table 1). In four studies, the Association Research Circulation Osseous (ARCO) classification was used (Table 2). In one study, the Steinberg classification was used (Table 2). The characteristics of patients included in the study are shown in Table 1. The average age of patients involved in this review was 34.48 years (range, 18–63 years). These studies investigated fibula allografting for treatment of ONFH in 394 hips with a mean follow-up of 49.06 months (range, 24–168 months). HHS was improved from 62.73 (average, preoperation) to 86.94 (average, latest follow-up) (Table 3). Radiographic progression was found in 33.66% of hips (range, 7.25–69%) (Table 4). The failure rate of head-saving surgery by THA was 14.5% (range, 3.44–34.5%) (Table 5). No patients had serious postoperative complications (Table 5).

**Table 1:** Characteristics of the included studies

Study	Study design	Total hips/stage of hips	Sex (F/M)	Mean age (year)	Mean follow-up (month)
Wei et al. 2011 [19]	Prospective, noncontrolled study	223 IIA, 10 IIB, 61 IIC, 63 IIIA, 89	61/101	33.5 (19–54)	24
Zeng et al. 2015 [20]	Retrospective, noncontrolled study	18 IIB, 5 IIC, 13	3/15	40.7 (22–49)	53.3
Wu et al. 2018 [21]	Retrospective, noncontrolled study	29 IIA, 9 IIB, 13 IIC, 4 IIIA, 3	7 (hips)/22 (hips)	38.9 (24–58)	168
Cao et al. 2018 [22]	Prospective randomized trial	II, 12 III, 17	10/17	37.6 (20–62)	41.1
		II, 13 III, 13	8/18	36.9 (18–60)	43.9
Feng et al. 2019 [23]	Retrospective cohort study	IIA, 12 IIB, 28 IIC, 19 IIIA, 4 IIIB, 4 IIIC, 2	39/30	31.5 (21–45)	84
		IIA, 3 IIB, 5 IIC, 5 IIIA, 18 IIIB, 18 IIIC, 20

**Table 2:** Classification systems used in the studies

System	Definition	
ARCO	Stage 0—bone biopsy positive, all imaging normal Stage I—normal findings on radiographs and abnormal MRI findings IA, < 15%; IB, 15–30%; IC, > 30% Stage II—abnormal X-ray findings, no femoral head collapse on X-ray and CT, and lesions were subdivided into medial, central, and lateral depending on the location of femoral head involvement IA, < 15%; IB, 15–30%; IC, > 30% Stage III—crescent sign, the lesion can be subdivided into medial, central, and lateral according to the position of femoral head Involvement IIIA: crescent sign, < 15% or collapse, < 2 mm IIIB: crescent sign, 15–30% or collapse, 2–4 mm IIIC: crescent sign, > 30% or collapse, > 4 mm Stage IV—osteoarthritic acetabular changes and cartilage changes	Wei et al. [19], Zeng et al. [20], Wu et al. [21], Feng et al. [23]
Steinberg	Stage 0—normal or nondiagnostic X-ray, bone scan, and MRI Stage I—normal X-ray, abnormal bone scan, and/or MRI Stage II—lucent and sclerotic changes in femoral head Stage III—subchondral collapse (crescent sign) without flattening Stage IV—flattening of femoral head Stage V—joint narrowing and/or acetabular changes Stage VI—advanced degenerative change	Cao et al. [22]

**Table 3:** Methods and outcomes of HHS in the studies

Study	Treatment	Assisted measures	Pre- vs. postop, Harris hip score	Results, excellent (*n*)/good (*n*)/rate (%)
Wei et al.	Fibula allograft	Autogenous bone graft	IIA, 72.5 vs. 96	6/4/100%
			IIB, 0.2 vs. 90	52/5/93.40%
			IIC, 64.3 vs. 80.3	20/17/58.70%
			IIIA, 51.5 vs. 85.4	30/47/86.50%
			Total, 61.2 vs. 87.5	108/73/81.10%
Zeng et al.	Fibula allograft	Allogeneic bone granule	IIB, 57.8 vs. 91.4	–
			IIC, 63.0 vs. 80.9	–
			Total, 61.6 vs. 83.8	–
Wu et al.	Two fibula allograft	–	Total, 50.3 vs. 76.1	–
Cao et al.	Fibula allograft	Multi-directional CD+ Autogenous bone graft	Total, 66.75 vs. 87.83	16/8/82.8%
	Fibula allograft	Single CD+ Autogenous bone graft	Total, 64.82 vs. 80.97	8/8/61.6%
Feng et al.	Fibula allograft	Allogeneic cancellous bone	Total, 70.7 vs. 92.4	–
	VGTF	Autogenous bone graft	Total, 58.1 vs. 84.2	–

*CD* core decompression, *VGTF* vascularized greater trochanter flap, *Rate* excellent and good rate

**Table 4:** Radiographic outcomes

Study	Radiographic progression (%)	Radiographic progression on stage (%)			
		I	II	III	IV
Wei et al.	42%				
Zeng et al.	22%		IIB, 0% IIC, 30.77%		
Wu et al.	69%				
Cao et al.	10.3%				
	26.9%				
Feng et al.	7.25%				
	36.23%

**Table 5:** Outcomes of the conversion rate to THA and complications

Study	Rate of conversion to THA	Rate of conversion to THA based on stage (ARCO)	Complication (*n*)
Wei et al.	19%	–	9, mild pyrexia 3, minor wound hematoma 3, deep infection
Zeng et al.	22%	IIB, 0% IIC, 22%	1, weakness of ankle and foot
Wu et al.	34.5%	IIA, 3.44% IIB, 17.24% IIC, 3.44% IIIA, 10.34%	–
Cao et al.	3.44%	–	–
	19.23%	–	1, subtrochanteric fracture
Feng et al.	4.35%	IIIB, 10.71%	–
	14.49%	IIC, 20% IIIB, 16.67% IIIC, 30%	1, superficial wound infection 2, greater trochanter pain

Wei et al. [19] treated 162 patients (223 hips: ARCO II, 52 hips; IIIA, 171 hips) using a fibula allograft and autogenous bone graft, which were followed for an average of 24 months. They found that the average HHS was improved from 61 points to 85.7, and the excellent and good rates were 93.3% in stage II and 87% in stage III. Imaging was stable without progression in 58% of patients. At the last follow-up, there were 49 (19%) failed hips that resulted in THA. Complications occurred in 15 patients, such as mild pyrexia, minor wound hematoma, and deep infection. All of these complications were cured non-operatively by medication.

Zeng et al. [20] studied a consecutive series of 18 patients with non-traumatic bilateral ONFH. One side was treated with core decompression (IIB, five hips; IIC, 13 hips) followed by allogeneic bone grafting and fibular allografting, and the other side with THA. The mean follow-up was 53.2 months. The mean HHS of 18 hip-preserving hips was 83.8 ± 17.9 points postoperatively compared with 61.6 ± 17.0 preoperatively (*P* < 0.05). Zeng et al. also quantified the results in terms of the visual analog scale (VAS) in 18 hip-preserving hips. The preoperative VAS score was 6.2 ± 2.0 points, whereas the postoperative score was 2.8 ± 2.3 points (*P* < 0.05). At the last follow-up, 14 hips (78%) achieved good outcomes, whereas four hips (22%) underwent THA as the disease progressed. No significant postoperative complications occurred in 18 hip-preserving hips.

Wu et al. [21] treated ONFH using two allografted fibulae that were dispersed slightly away to support the on weight-bearing area. Through long-term follow-up (average, 14 years; range, 1–21 years), they observed that the success rate of preservation of the femoral head was 65.5%, the radiological success rate was 31%, and the mean HHS improved from 50.3 to 76.1. They concluded that gender, ONFH stage, and necrotic index were independent risk factors for conversion to THA.

Cao et al. [22] conducted a prospective randomized trial in which the experimental group underwent fibula allografting and autologous cancellous bone grafting, whereas the control group underwent fibula allografting only. After a mean 42.7-month follow-up, although the clinical efficacy (HHS and VAS) of the two groups showed a marked improvement (*P* < 0.001), the experimental group had better efficacy than the control group (*P* < 0.01). Although no significant statistical difference was observed in imaging progression between the experimental group (10.3%) and control group (26.9%), the number of improved hips in the experimental group was higher than that in the control group. THA was performed in one hip of the experimental group and in five hips of the control group. Only one patient in the control group had a subtrochanteric fracture and no other complications occurred.

Feng et al. [23] performed a retrospective cohort study of patients with two sides of ONFH for a mean of 7.0 years. One side was treated with a fibula allograft and cancellous bone (group A), and the other side with vascularized greater trochanter flap autografting (group B). In group A, the HHS improved from preoperative 70.7 ± 3.5 points (range 64–76 points) to postoperative 92.4 ± 4.0 points (range 80–98 points) (*P* < 0.01). The VAS was also included in their ratings, which decreased from 4.8 ± 1.2 points (range 3–9 points) to 1.1 ± 1.0 points (range 0–4 points) (*P* < 0.01). Only five hips in group A showed progression in imaging. Three hips at stage IIIB in group A needed a THA conversion at mean postoperative 5.6 years (range 4–7 years). Three hips at stage IIIB underwent THA at mean postoperative 5.6 years (range 4–7 years) after surgery. No postoperative complications occurred in group A.

## Discussion

The purpose of our review was to find evidence in articles to evaluate whether a fibula allograft was an effective treatment for early-stage ONFH. In this review, after searching three databases and other resources, we identified five articles that determined the effect of a fibula allograft on treating ONFH. All fibula allografts included in the studies were applied for ONFH treatment by “Phemister technology.”

In 1949, Phemister first proposed the technique of non-vascularized bone transplantation for the treatment of early femoral head necrosis, namely the “Phemister technique” [21, 24].The technique uses an 8- or 10-mm diameter trephine through the lateral femoral neck of the greater trochanter to the necrotic area of the femoral head. The dead bone is scraped with a special tool and then inserted into the cortical bone to prevent the femoral head from collapsing. A fibula allograft has abundant sources without donor site morbidity, low immunogenicity, a strong osteogenic ability, and the same elastic modulus as autogenous bone with a certain mechanical strength and supporting effect [19, 25].

In this review, two studies reported the results of fibula allografting and autogenous cancellous bone grafting for ONFH [19]. One article was related to treatment of ONFH using a fibula allograft with a cancellous bone allograft, one with two fibula allografts, and one included a simple fibula graft. Different from most other studies [26–28], Wu et al. used two fibular allografts tapping into the femoral head deeply to the subchondral bone [21]. However, they did not perform a biomechanical analysis of the optimal number of grafts and position for structural support. Nonetheless, they believe that using two fibular allografts might provide more mechanical strength than a single allograft and without increasing the risk of fracture. Cao et al. found that using a multi-directional core decompression apparatus combined with a fibula allograft and autologous cancellous bone implantation was better than traditional methods. Feng et al. [20] demonstrated that a fibula allograft plus a cancellous bone allograft was more effective than vascularized greater trochanter flap autografting for the treatment of ARCO stage II ONFH. In comparison with vascularized fibular grafting, non-vascularized fibula allograft transplantation was less invasive and achieved similar clinical effects [29–31].

There are some limitations in this study. The number of articles included in the study was small, and all studies were single-center studies. Most studies were retrospective with a low level of evidence. Although the included patients were treated with fibula allografts for ONFH, their surgical procedures were not identical with different follow-up times.

## Conclusion

In summary, after analyzing the relevant data, we found that allogeneic fibula transplantation is an effective method to treat early-stage ONFH, which improved hip functions, delayed the development of ONFH, and lowered the rate of THA. Allogeneic fibula transplantation for the treatment of early-stage ONFH is simple, has less postoperative complications, satisfactory clinical effects, and is worthy of promotion as a clinical therapy for ONFH.

## Data Availability

All data and materials used to support the findings of this study are included within the article.

## Abbreviations

ONFH: Osteonecrosis of the femoral head
THA: Total hip arthroplasty
HHS: Harris hip scoring
ARCO: Association Research Circulation Osseous
VAS: Visual analog score
